# Ocular Manifestations in Pregnancy-Induced Hypertension at a Tertiary Level Hospital in Karnataka, India

**DOI:** 10.7759/cureus.34887

**Published:** 2023-02-12

**Authors:** Chethana Warad, Bharat Midha, Utkarsh Pandey, Pavuluri Sivakrishna, Arpit Jain, Bhoomi Bagadia, Vatsal Makhija, Bhargavi Pravin Patil, Srivardhan Cheguri, Bhagyajyoti B K

**Affiliations:** 1 Ophthalmology, KLE Jawaharlal Nehru Medical College, Belagavi, IND; 2 Medicine, KLE Jawaharlal Nehru Medical College, Belagavi, IND; 3 Emergency Medicine, All India Institute of Medical Sciences, Delhi, IND

**Keywords:** ocular, fundus, retina, blood pressure, gestational age, eclampsia, pre-eclampsia, pregnancy induced hypertension (pih), pregnancy

## Abstract

Introduction

Pregnancy-induced hypertension (PIH) is a hypertensive disorder in pregnancy that occurs after 20 weeks of pregnancy in the absence of previously known hypertension.

PIH is a common and serious complication accompanying pregnancy. Pre-eclampsia and eclampsia are multisystem disorders that can involve end organs like kidneys, liver, eyes, haematopoietic system and placenta. Though ocular involvement is not uncommon in PIH, ocular examination is not always done in all cases of PIH. Timely detection of changes in retinal vasculature can be a hint to the underlying changes in the vascular system of the various end organs of the human body including placental circulation. Adequate management of PIH is very important for both fetal and maternal well-being.

Aim

To evaluate the ocular manifestations in women affected by PIH (mild pre-eclampsia, severe pre-eclampsia and eclampsia) presenting to a tertiary-level hospital.

Methodology

This was a hospital-based cross-sectional study carried out for a period of one year at a tertiary-level hospital. A total of 120 subjects diagnosed as cases of pre-eclampsia/eclampsia admitted to the eclampsia ward of the obstetric unit formed the study population. After taking history, a detailed ocular examination was done for all patients and the findings were noted.

Results

The mean age of the study population was 31.91 ± 4.38 years (range 21 to 39 years). The mean gestational age was 30.89 ± 3.98 weeks. Fifty-three (44.17%) were primigravida, 64 (53.33%) were multiparous, and three (2.5%) were grand multiparous. Sixty-two (51.67%) had mild pre-eclampsia, 50 (41.67%) had severe pre-eclampsia and eight (6.67%) had eclampsia. The mean systolic blood pressure (SBP) and diastolic blood pressure (DBP) recorded in the study were 155.32 ± 11.89 mmHg and 104.3 ± 11.41 mmHg respectively. Ocular symptoms were present in 43 (35.83%) participants. Blurring of vision (19.17%) was the commonest ocular symptom observed in the study population followed by photopsia (13.33%), diplopia (9.17%), intermittent loss of vision (5.83%), ocular pain (6.67%), and scotoma (1.67%). Systemic symptoms included headache (11.67%), epigastric pain (3.33%), and nausea (5%). Anterior segment findings like conjunctival congestion, lid edema, and subconjunctival hemorrhage each accounted for 1.67% of the study population. Fundal changes were present in 33.33% of cases. Arteriolar narrowing was the commonest fundal finding amounting to 15.83%, followed by arteriovenous (AV) crossing changes also in 15.83%, cotton wool spots in 5.83%, retinal haemorrhages in 8.33%, papilledema in 2.5%, and choroidal infarcts in 1.67% participants. Grade 1 hypertensive retinopathy was observed in 15.83% of participants, grade 2 in 8.33% of participants, grade 3 in 6.67% of participants and grade 4 in 2.5% of participants. The mean SBP and mean DBP were high among those with fundal changes (163.35 ± 10.25 mmHg and 111.15 ± 10.29 mmHg) compared to those without fundal changes (151.3 ± 10.58 mmHg and 100.88 ± 10.41 mmHg). This was statistically significant. Proteinuria showed significant correlation with retinal changes.

Conclusion

The retinal vasculature changes correlate with the severity of hypertension, hence, it is very important to seek ophthalmologic opinion for evaluation, diagnosis and prompt management of PIH.

## Introduction

Pregnancy is indeed a challenge to the human body and is associated with many physiological and pathological changes. Pregnancy-induced hypertension (PIH) is one of the most common complications of pregnancy and if not recognized early and treated appropriately, can seriously affect maternal and child health. The estimated incidence of eclampsia in European countries is one in 2000 pregnancies [1]. In India, the incidence of eclampsia ranges from 0.17 to 3.7% [2-4], and maternal mortality from 2.2 to 23% of all women with eclampsia [3-6]. Young primigravidas, ignorance, poverty, lack of resources, and lack of adequate prenatal care in remote areas are the main social factors contributing to the high incidence of eclampsia in India. Undertreated eclampsia accounts for 75% of all maternal deaths [7] and causes irreversible blindness in 1-3% of affected cases [8]. Ocular manifestations affect up to 50% of patients with eclampsia and 25% of patients with severe pre-eclampsia [9]. Blurred vision, intermittent vision loss, diplopia, photopsia, and scotoma are the most common symptoms observed in cases of pre-eclampsia and eclampsia. Complete vision loss is uncommon but can occur due to visual cortex involvement, retinal detachment, or optic nerve atrophy. Focal or generalized arteriolar stenosis (70%) is the primary response to systemic arterial hypertension and has been described as the most common presentation in patients with PIH.

Pre-eclampsia/eclampsia is a multi-system disease that can affect end organs such as kidneys, liver, eyes, hemopoietic system, and placenta. Retinal involvement is fairly common but not always investigated.

It’s a known fact that the eye is a unique structure, wherein blood vessels can be visualized directly and non-invasively through the technique of fundoscopy. Fundoscopy is not only useful in assessing the condition of the eye and the effects of high blood pressure on the blood vessels of the retina but also to understand the effect of high blood pressure on other organs of the body including placental circulation and fetal health [10]. Therefore, it is very important that the attending physician/obstetrician seeks an ophthalmological examination in each and every case of PIH.

The current study aimed to evaluate the ocular manifestations in women affected by pregnancy-induced hypertension (mild and severe pre-eclampsia and eclampsia) presenting to a multi-disciplinary tertiary-level charitable teaching hospital in Karnataka, India.

## Materials and methods

This was a hospital-based descriptive cross-sectional study, conducted for a period of one year from January 2022 to December 2022 in a tertiary-level multi-disciplinary hospital affiliated with a prestigious medical college in north-western Karnataka, India. Data were collected from the obstetric eclampsia ward.

Inclusion criteria

All patients diagnosed with pre-eclampsia or eclampsia, admitted to the obstetrics eclampsia ward and willing to participate in the study, were included.

Exclusion criteria

Patients with a history of chronic hypertension, pre-existing diabetes mellitus, renal disease, hematological disease, infectious disease, thyroid disease, HIV, hepatitis B surface antigen (HBsAg), prior ocular pathologies, and those who didn’t wish to participate in the study were excluded from the study.

A total of 120 subjects formed the study population after applying inclusion and exclusion criteria.

Study procedure

After a brief obstetric history, an ocular history was also recorded. Case notes were reviewed and obstetric details such as age, parity, gestational age, blood pressure, and proteinuria scores were recorded. The ophthalmological examination included visual acuity testing using a Snellen’s chart and a pinhole was used to assess the visual acuity. The intraocular pressure was measured under topical anesthesia with Tonopen. The anterior segment examination was done using a torch and handheld portable slit lamp. Fundoscopy was performed with a head-mounted indirect ophthalmoscope (Appasamy, Chennai, India) with 20 dioptres after pupil dilatation with tropicamide 0.8% and phenylephrine 5%. All the data collected were documented in the data sheet.

Pregnancy-induced hypertension severity was classified into pre-eclampsia (mild and severe) and eclampsia, as described in the following paragraph. Mild pre-eclampsia was defined as a blood pressure greater than 140/90 mmHg, + proteinuria, and/or mild leg edema. Severe pre-eclampsia was defined as blood pressure greater than 160/110 mmHg, ++ or +++ proteinuria, headache, cerebral or visual disturbances, epigastric pain, impaired liver function tests, and increased serum creatinine. Eclampsia was defined as severe pre-eclampsia superseded by convulsions. For purpose of dipstick quantification of proteinuria, a single '+' correlates to 30mg/dL, '++' correlates to 100mg/dL, and '+++' correlates to 300mg/dL [11,12].

Ethical clearance for the study was obtained from the institutional review board of Jawaharlal Nehru Medical College (JNMC) prior to study initiation (Ref No. MDC/JNMCIEC/373 dated 20th January 2021).

Statistical analysis

Data were analyzed using statistical software R version 4.2.1 and Microsoft Excel (Redmond, WA, USA). Categorical variables are given in the form of frequency tables. Continuous variables are given in mean ± SD; median (min, max) form. Chi square test was used to check the association between categorical variables. Two-sample t-tests were used to compare means over fundus changes. Mann Whitney U test was used to compare the distribution of variables over fundus changes. One-way ANOVA was used to compare the means over hypertensive retinopathy. Kruskal Wallis test was used to compare the distributions of variables over hypertensive retinopathy. P-value less than or equal to 0.05 indicates statistical significance.

## Results

The distribution of subjects according to different variables like age, gestational age, parity, pregnancy-induced hypertension, blood pressure, proteinuria, ocular symptoms, systemic symptoms, anterior segment findings, fundus changes, and hypertensive retinopathy classification is shown in Table 1 and depicted in consecutive figures and accompanying text.

**Table 1:** Distribution of subjects according to different variables

Variables	Sub Category	Number of subjects (%)
Age (years)	21-25	7 (5.83%)
	26-30	41 (34.17%)
	31-35	37 (30.83%)
	36-40	35 (29.17%)
	Mean ± SD	31.91 ± 4.38
	Median (Min, Max)	32 (21, 39)
Gestational age (weeks)	<28	21 (17.5%)
	28-34	66 (55%)
	34-37	24 (20%)
	≥37	9 (7.5%)
	Mean ± SD	30.89 ± 3.98
	Median (Min, Max)	31 (23, 40)
Parity	Primigravida	53 (44.17%)
	Multigravida	64 (53.33%)
	Grand Multigravida	3 (2.5%)
Status	Eclampsia	8 (6.67%)
	Moderate Preeclampsia	62 (51.67%)
	Severe Preeclampsia	50 (41.67%)
Systolic Blood Pressure (mmHg)	Mean ± SD	155.32 ± 11.89
	Median (Min, Max)	154 (140, 180)
Diastolic Blood Pressure (mmHg)	Mean ± SD	104.3 ± 11.41
	Median (Min, Max)	100 (90, 124)
Proteinuria	+	60 (50%)
	++	23 (19.17%)
	+++	37 (30.83%)
Ocular symptoms	Blurred Vision	23 (19.17%)
	Photopsia	16 (13.33%)
	Diplopia	11 (9.17%)
	Headache	1 (0.83%)
	Intermittent Loss of Vision	7 (5.83%)
	Ocular pain	8 (6.67%)
	Scotoma	2 (1.67%)
	None	76 (63.33%)
Systemic symptoms	Epigastric pain	4 (3.33%)
	Headache	13 (10.83%)
	Nausea	6 (5%)
	None	98 (81.67%)
Anterior segment findings	Chemosis	2 (1.67%)
	Conjunctival congestion	2 (1.67%)
	Lid edema	2 (1.67%)
	Subconjuctival Hemorrhage	2 (1.67%)
	None	112 (93.33%)
Fundus changes	Arteriolar narrowing	19 (15.83%)
	Arteriovenous crossing changes	19 (15.83%)
	Cotton wool spots	7 (5.83%)
	Retinal haemorrhages	10 (8.33%)
	Papilledema	3 (2.5%)
	Choroidal infarcts	2 (1.67%)
	None	80 (66.67%)
Hypertensive retinopathy	Grade 1	19 (15.83%)
	Grade 2	10 (8.33%)
	Grade 3	8 (6.67%)
	Grade 4	3 (2.5%)
	Nil	80 (66.67%)

**Figure 1:**
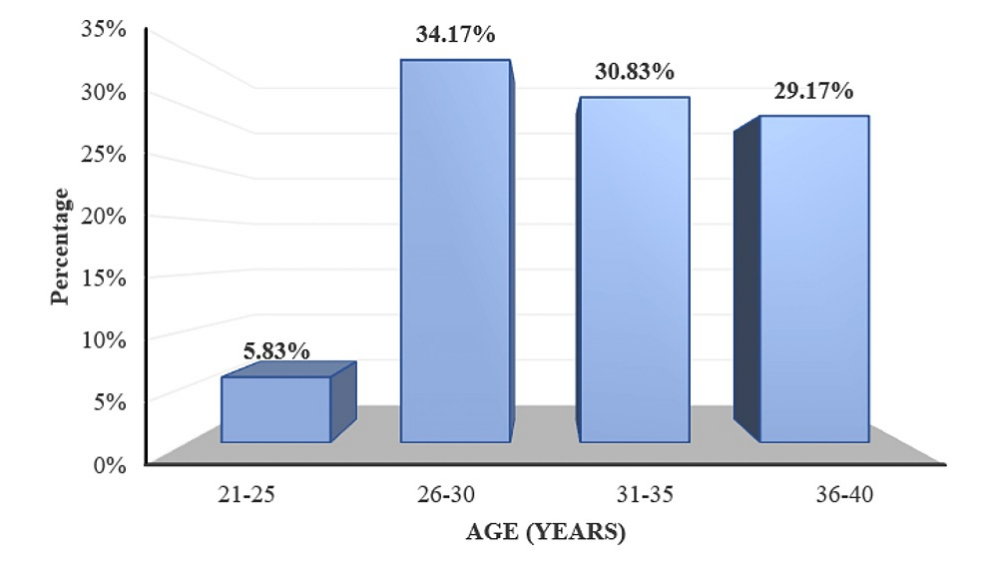
Distribution of subjects according to age

A total of 120 pregnant women diagnosed with PIH (mild pre-eclampsia/severe pre-eclampsia/eclampsia) were tested. The mean age of the study population was 31.91 ± 4.38 years (range 21 to 39 years) (Figure 1). 

**Figure 2:**
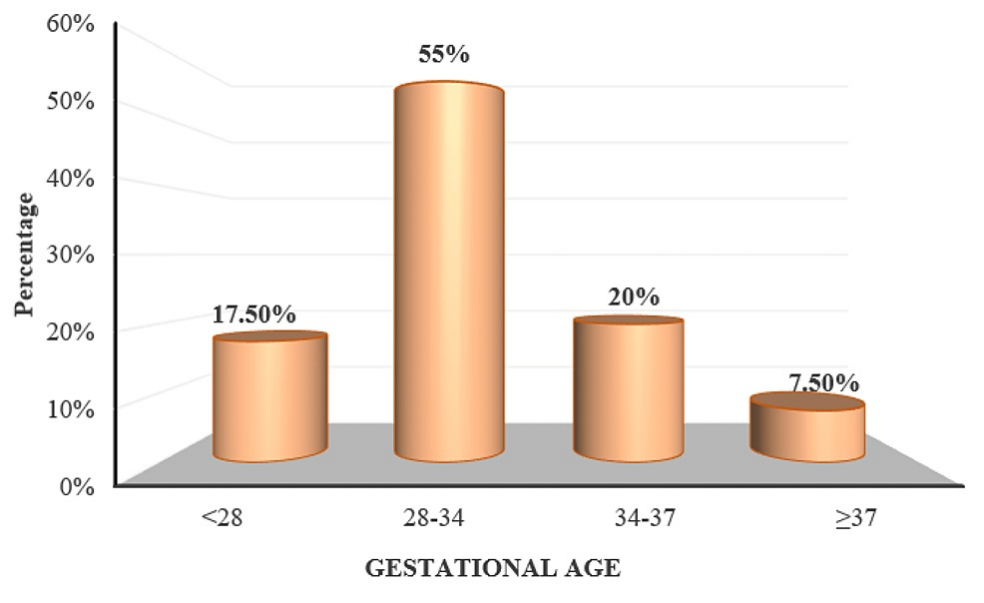
Distribution of subjects according to gestational age

Twenty-one (17.5%) participants were at <28 weeks of gestation, 66 (55%) participants were between 28 and 34 weeks of gestation, 24 (20%) were between 34 and 37 weeks of gestation and nine (7.5%) were equal to or more than 37 weeks of gestation (Figure 2). The mean gestational age was 30.89 ± 3.98 weeks.

**Figure 3:**
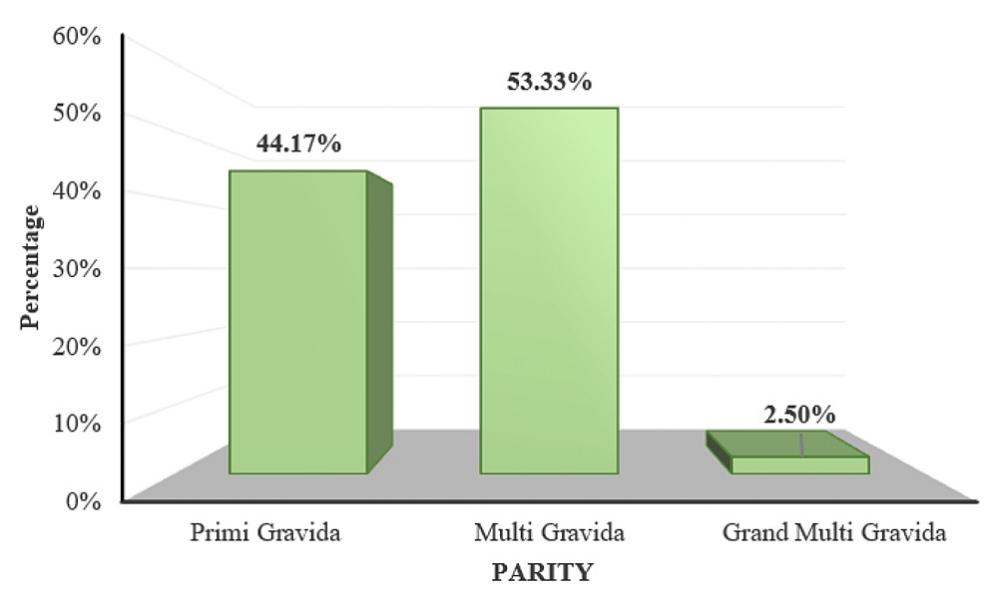
Distribution of subjects according to parity

Fifty-three (44.17%) participants were primiparous, 64 (53.33%) participants were multiparous, and three (2.5%) participants were grand multiparous (Figure 3). 

**Figure 4:**
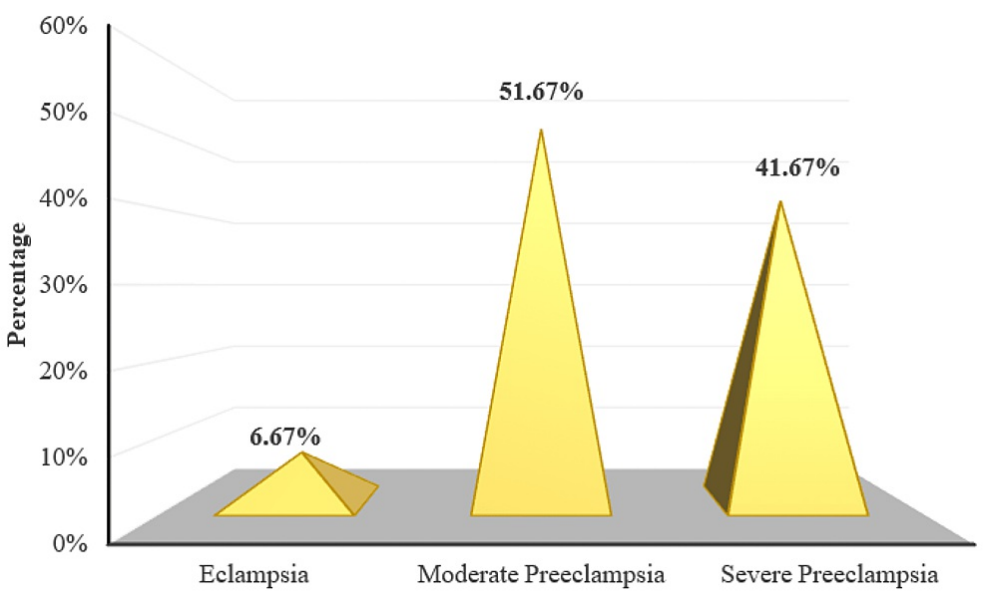
Distribution of subjects according to status

Sixty-two (51.67%) participants had mild pre-eclampsia, 50 (41.67%) participants had severe pre-eclampsia and eight (6.67%) participants had eclampsia (Figure 4).

**Figure 5:**
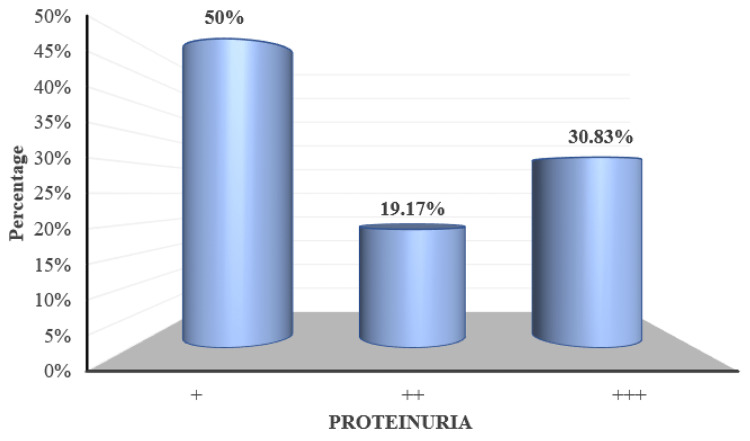
Distribution of subjects according to proteinuria

'+' proteinuria was recorded in 60 (50%) of the participants, '++' proteinuria was recorded in 23 (19.17%) of participants and '+++' proteinuria was recorded in 37 (30.83%) of participants (Figure 5).

**Figure 6:**
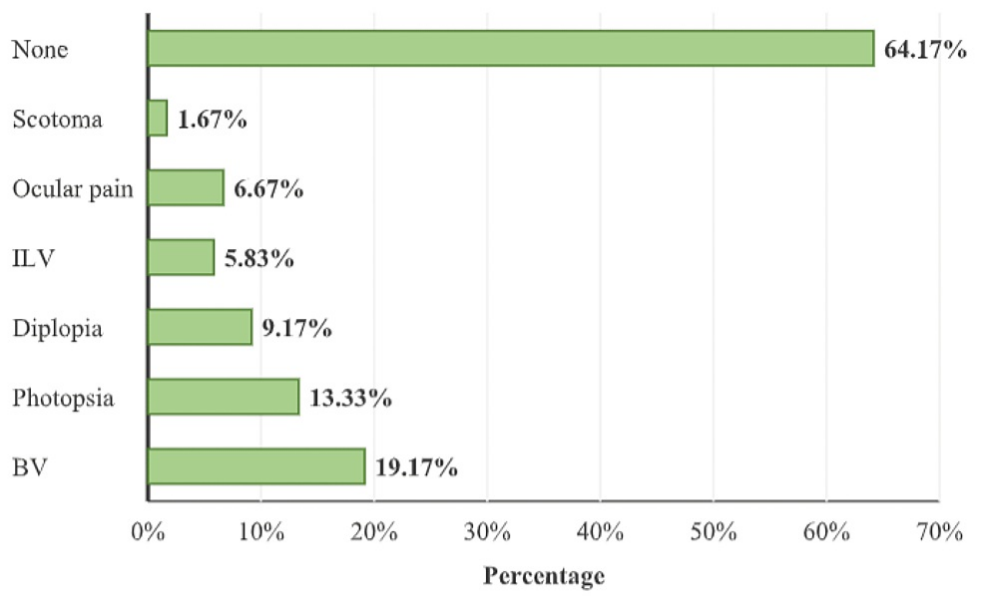
Distribution of subjects according to ocular symptoms ILV: Intermittent Loss of Vision; BV: Blurred Vision

Ocular symptoms were seen in 43 (35.83%) participants. Blurred vision (19.17%) was the most common ocular symptom observed in the study population followed by photopsia (13.33%), diplopia (9.17%), intermittent loss of vision (ILV) (5.83%), ocular pain (6.67%), and scotoma (1.67%) (Figure 6). 

**Figure 7:**
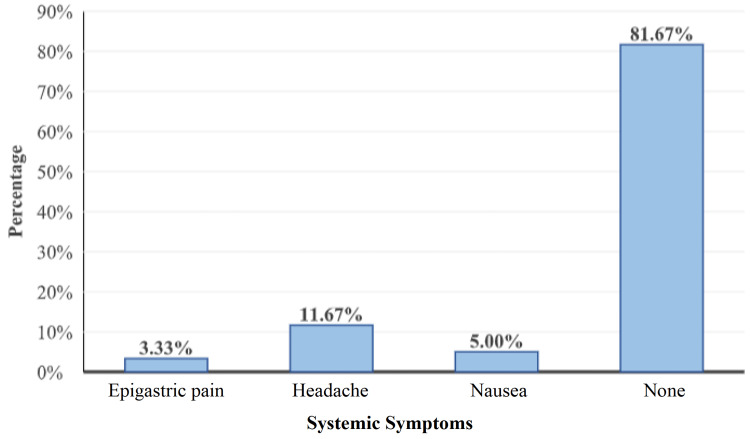
Distribution of subjects according to systemic symptoms

Systemic symptoms were seen in 18.33% of the participants. Headache was seen in 13 (11.67%) participants, epigastric pain was seen in four (3.33%) participants, and nausea was seen in six (5%) participants (Figure 7).

**Figure 8:**
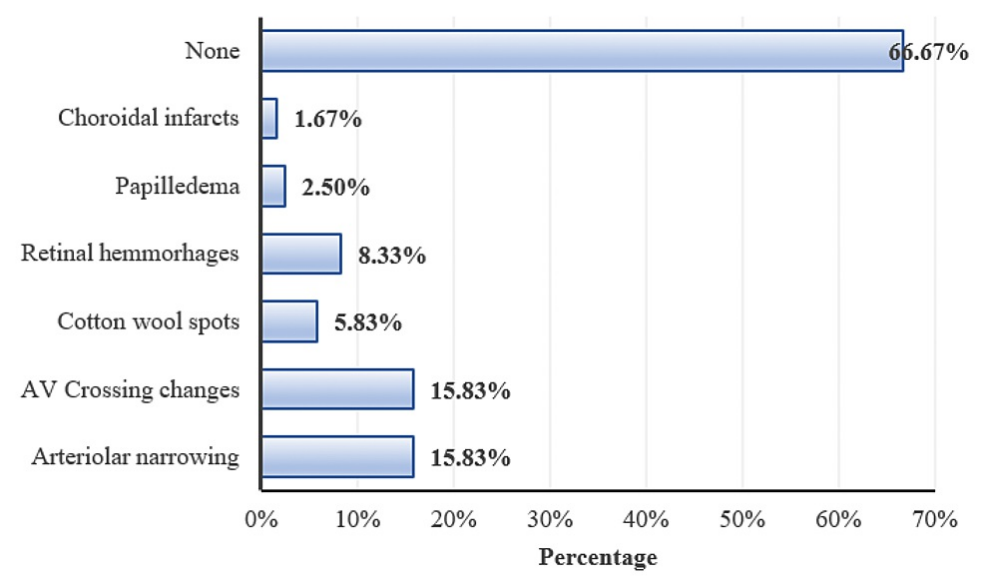
Distribution of subjects according to fundus changes AV: Arteriovenous

Fundal changes were present in 40 (33.33%) cases. Arteriolar narrowing was the most common retinal finding amounting to 19 (15.83%) participants, followed by arteriovenous crossing changes in 19 (15.83%) participants, cotton wool spots in seven (5.83%) participants, retinal hemorrhages in 10 (8.33%) participants, papilledema in three (2.5%) participants, choroidal infarcts in two (1.67%) participants (Figure 8). Anterior segment findings such as conjunctival congestion, lid edema, and subconjunctival hemorrhage each accounted for 1.67% of the study population (Figure 8).

**Figure 9:**
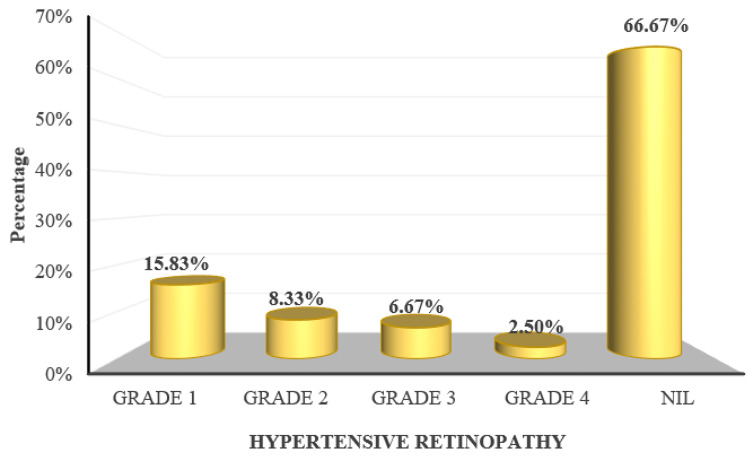
Distribution of subjects according to hypertensive retinopathy as per the Keith-Wagener-Barker grading system

The mean systolic blood pressure and diastolic blood pressure recorded in the study were 155.32 ± 11.89 mmHg and 104.3 ± 11.41 mmHg respectively. Following the Keith-Wagener-Barker grading system, grade 1 hypertensive retinopathy was observed in 19 (15.83%) participants, grade 2 in 10 (8.33%) participants, grade 3 in eight (6.67%) participants, and grade 4 in three (2.5%) participants (Figure 9). 

Table 2 and the proceeding figures, along with text, show comparisons of age, gestational age, parity, PIH status, systolic and diastolic blood pressure, and proteinuria with fundus changes.

**Table 2:** Comparison of different variables over fundus changes SBP: Systolic Blood Pressure; DBP: Diastolic Blood Pressure; MC: Chi Square test with Monte Carlo simulation; MW: Mann Whitney U test; t: Two-sample t-test. '*' after the p-value denotes statistical significance.

Variables	Sub Category	Fundus changes		p-value
		No	Yes	
Age (years)	21-25	5 (6.25%)	2 (5%)	0.1129^MC^
	26-30	32 (40%)	9 (22.5%)	
	31-35	25 (31.25%)	12 (30%)	
	36-40	18 (22.5%)	17 (42.5%)	
	Mean ± SD	31.31 ± 4.29	33.1 ± 4.35	0.0282^MW^*
	Median (Min, Max)	31 (21, 39)	34.5 (22, 38)	
Gestational age (weeks)	<28	15 (18.75%)	6 (15%)	0.7801^MC^
	28-34	45 (56.25%)	21 (52.5%)	
	34-37	14 (17.5%)	10 (25%)	
	≥37	6 (7.5%)	3 (7.5%)	
	Mean ± SD	30.68 ± 4.01	31.32 ± 3.94	0.4014^t^
	Median (Min, Max)	30 (23, 40)	32 (24, 40)	
Parity	Primigravida	40 (50%)	13 (32.5%)	0.0825^MC^
	Multigravida	37 (46.25%)	27 (67.5%)	
	Grand Multigravida	3 (3.75%)	0	
Status	Eclampsia	1 (1.25%)	7 (17.5%)	< 0.001^MC^*
	Moderate Preeclampsia	55 (68.75%)	7 (17.5%)	
	Severe Preeclampsia	24 (30%)	26 (65%)	
Systolic Blood Pressure (mmHg)	Mean ± SD	151.3 ± 10.58	163.35 ± 10.25	< 0.001^MW^*
	Median (Min, Max)	148 (140, 170)	165 (140, 180)	
Diastolic Blood Pressure (mmHg)	Mean ± SD	100.88 ± 10.41	111.15 ± 10.29	< 0.001^MW^*
	Median (Min, Max)	96 (90, 120)	114 (90, 124)	
Proteinuria	+	54 (67.5%)	6 (15%)	< 0.001^MC^*
	++	13 (16.25%)	10 (25%)	
	+++	13 (16.25%)	24 (60%)	

**Figure 10:**
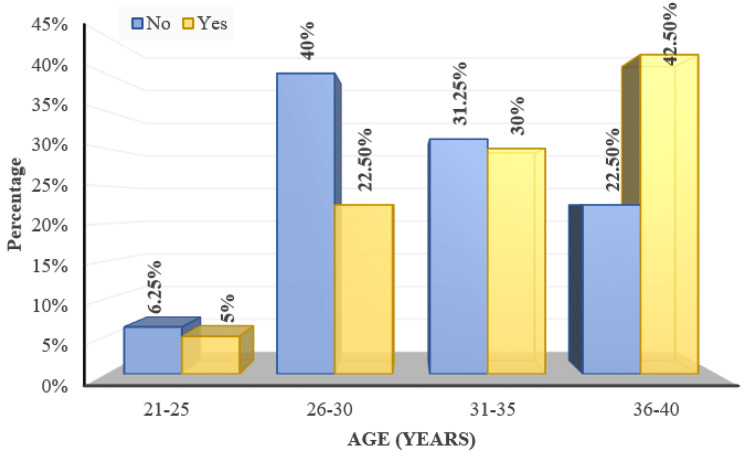
Distribution of age over fundus changes 'Yes/No' indicates the presence of fundal changes

The mean age of subjects with fundal change was 33.1 ± 4.35 years and the mean age of subjects without fundal change was 31.31 ± 4.29 years. This was statistically significant (p=0.02) from Mann Whitney U test (Figure 10). 

**Figure 11:**
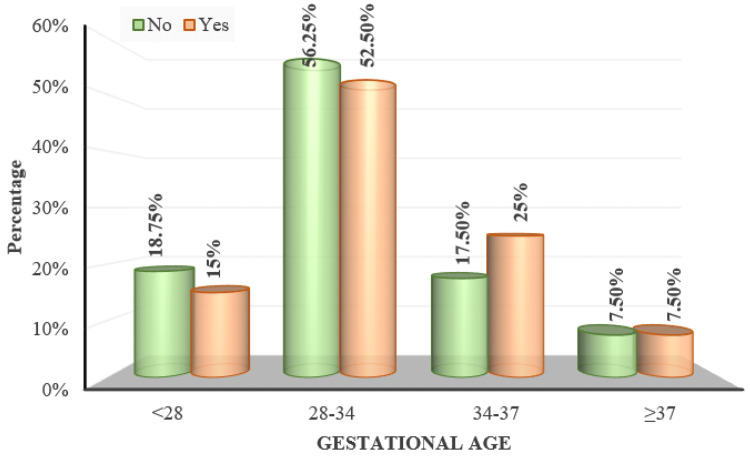
Distribution of gestational age over fundus changes 'Yes/No' indicates the presence of fundal changes

The mean gestational age of the subjects with fundal changes was 31.32 ± 3.94 weeks and without fundus changes was 30.68 ± 4.01 weeks which was statistically not significant according to the two-sample t-test (p=0.4) (Figure 11). 

**Figure 12:**
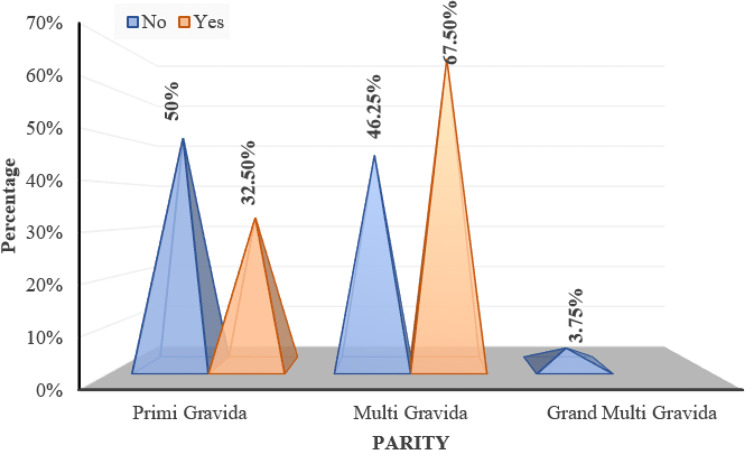
Distribution of parity over fundus changes 'Yes/No' indicates the presence of fundal changes

As per the Chi Square test with Monte Carlo simulation, parity did not show a significant correlation with the presence of fundal changes (p=0.085) (Figure 12).

**Figure 13:**
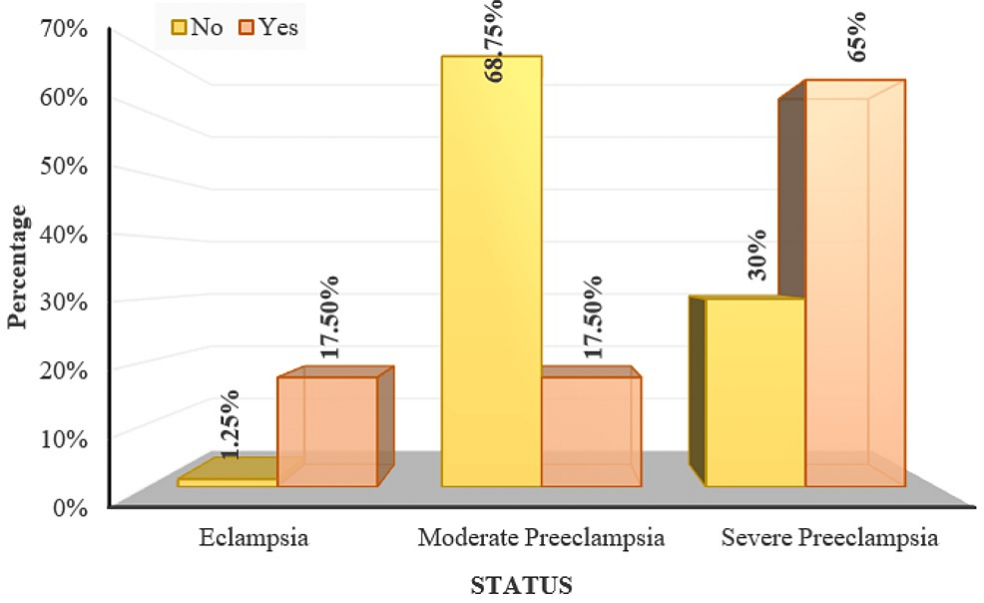
Distribution of pregnancy-induced hypertension (PIH) severity over fundus changes 'Yes/No' indicates the presence of fundal changes

As per the Chi Square test with Monte Carlo simulation, PIH status shows a significant correlation with the presence of fundal changes (p<0.001) (Figure 13). The mean systolic blood pressure (SBP) and mean diastolic blood pressure (DBP) were high among those with fundal changes (163.35 ± 10.25 mmHg and 111.15 ± 10.29 mmHg) compared to those without fundal changes (151.3 ± 10.58 mmHg and 100.88 ± 10.41 mmHg). This was statistically significant as per the Mann Whitney U Test (p<0.001).

**Figure 14:**
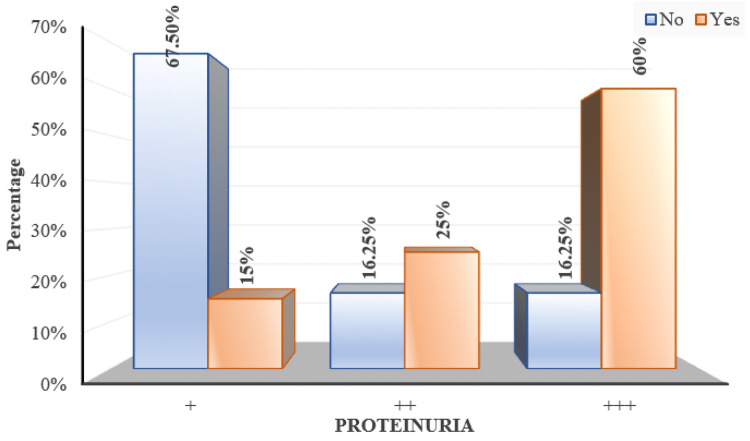
Distribution of proteinuria over fundus changes 'Yes/No' indicates the presence of fundal changes

As per the Chi Square test with Monte Carlo simulation, proteinuria (measured by urine albumin by dipstick method) showed significant correlation with retinal changes (p<0.001) (Figure 14).

## Discussion

Pregnancy is a physiological state with many changes in the physiological and biochemical parameters of the body. Pregnancy-induced hypertension is a difficult stigma in obstetrics and a leading cause of maternal and perinatal mortality [13-18]. PIH is a hypertensive disorder in pregnancy that occurs in the absence of other causes of elevated blood pressure (140/90 mmHg) or a rise of 30 mmHg of diastolic blood pressure taken on two occasions after rest, in combination with generalized edema and/or proteinuria. When there is significant proteinuria it is termed pre-eclampsia; seizures or coma as a consequence of PIH is termed eclampsia [19].

Changes in the eye during pregnancy are common. Most of these are benign physiological responses to metabolic, hormonal, and immunological changes to adapt the gestational product, there are some variations in the retinal vasculature that can be used as a good guide to assessing the severity of hypertension and for differentiating between chronic hypertension and PIH.

The aim of the current study was to determine the prevalence of retinal changes in PIH and the association of retinal changes with blood pressure, proteinuria, and disease severity. The majority of the patients in the present study were in the age group of 26 to 30 years with a mean age of 31.91 ± 4.38 years, which was comparable with the mean ages of the studies by Reddy et al. [20] in the Malaysian population and Indu et al. [10] in the South Indian population. The commonly observed gestational age group was between 28 weeks to 34 weeks. In the current study, the number of multiparous women exceeded the number of primigravidas. Parity and gestational age showed no correlation with retinal findings in the present study.

Of 120 subjects, 43 (35.83%) had ocular symptoms, 23 (19.17%) had systemic symptoms and 40 (33.33%) had retinal changes. Blurred vision was the most common ocular symptom, followed by photopsia. Headache (11.67%), epigastric pain (3.33%), and nausea (5.0%) were the systemic symptoms observed in our study. Our results were consistent with a study done on the Indian population by Indu et al. [10] and Bhandari et al. [21]. 

In the present study arteriolar narrowing (15.3%) and arteriovenous crossing changes (15.3%) were the most common retinal changes noted followed by retinal hemorrhages (8.33%), cotton wool spots (5.83%), papilledema (2.5%), and choroidal infarct (1.67%). Jaffee et al. [22] had a similar finding where they found a statistically significant correlation between reduction in arteriole-to-vein ratio, number of focal restrictions, and diagnosis of severe pre-eclampsia. A study done by Bhandari et al. [21] found arteriolar attenuation in 44% of cases followed by macular edema in 12% of cases and exudative retinal detachment in 7% of cases.

Another study done by Indu et al. [10] found that 40% of cases had arteriolar narrowing followed by 12.7% of cases of macular edema, 4% of cases of arteriovenous crossing changes, 1.3% of cases with cotton wool spots, 2% of cases with retinal hemorrhages and 1.3% of cases with choroidal infarcts. Our results contrast with a study done among the Tanzanian population at Muhimbili National Hospital [23] which found maximum cases of papilloedema (28%) followed by retinal oedema (19.5%) and cotton wool spots (18.6%), arteriolar narrowing in 17.7% and macular edema in 5.3%.

In the current study we found that subjects with retinal changes had higher systolic and diastolic blood pressure (p<0.001) than those without fundal changes. Similarly significant increase in urine albumin was noted with an increase in the severity of PIH and significant increase in the mean systolic and diastolic blood pressure was seen with the increase in severity of retinopathy among the study subjects in a study done by Devaru et al. [24]. Also studies done by Bhandari et al. [21], Indu et al. [10], and Mackensen et al. [25] mentioned that severe grades of retinopathy were associated with elevated levels of blood pressure.

Also in the present study we noticed that higher grades of proteinuria had more fundus involvement (p<0.001) which was in agreement with findings of studies done by Devaru et al. [24] and Bhandari et al. [21] and in contrast with the findings of studies done by Mithila et al. [26], Indu et al. [10] and Uma et al. [27] who mentioned that there is no relation with proteinuria and occurrence of fundus changes.

Rare ocular complications of pre-eclampsia and eclampsia include choroidal infarcts, papillophlebitis, retinal artery and vein occlusion, ischaemic optic neuropathy, optic atrophy, optic neuritis, thrombosis of the central retinal artery and Purtscher-like retinopathy [28-30]. In the present study we witnessed two subjects (1.67%) with choroidal infarcts; the rest of these rare ocular complications were fortunately not seen.

## Conclusions

Ocular involvement is quite often among pregnant women with pre-eclampsia and eclampsia, so it is very important for obstetricians to be aware of the various ocular conditions associated with pregnancy-induced hypertension and to seek for routine fundal examinations by ophthalmologists in all pre-eclampsia and eclampsia patients in view of fetal and maternal well-being. Early fundus screening in patients with PIH not only helps detect changes in the retina but also serves as a guide to detect similar changes in other organs of the body including the placental circulation, greatly assisting diagnosis and prompt treatment.
